# Nanoparticle formulation increases *Syzygium cumini* antioxidant activity in *Candida albicans*-infected diabetic rats

**DOI:** 10.1080/13880209.2017.1283338

**Published:** 2017-02-13

**Authors:** Paula E. R. Bitencourt, Lariane O. Cargnelutti, Carolina S. Stein, Raquel Lautenchleger, Luana M. Ferreira, Manuela Sangoi, Laura Denardi, Raphaela M. Borges, Aline Boligon, Rafael N. Moresco, Letícia Cruz, Régis A. Zanette, Sydney H. Alves, Maria Beatriz Moretto

**Affiliations:** a Departamento de Análises Clínicas e Toxicológicas, Centro de Ciências da Saúde, Programa de Pós-Graduação em Ciências Farmacêuticas, Universidade Federal de Santa Maria, Santa Maria, Brazil;; b Departamento de Farmácia Industrial, Centro de Ciências da Saúde, Programa de Pós-Graduação em Ciências Farmacêuticas, Universidade Federal de Santa Maria, Santa Maria, Brazil;; c Departamento de Microbiologia, Centro de Ciências da Saúde, Programa de Pós-Graduação em Ciências Farmacêuticas, Universidade Federal de Santa Maria, Santa Maria, Brazil;; d Programa de Pós-Graduação em Ciências Biológicas: Farmacologia e Terapêutica, Universidade Federal do Rio Grande do Sul, Porto Alegre, Brazil

**Keywords:** Advanced oxidation protein product, N-acetyl-β-d-glucosaminidase, TBARS, streptozotocin, diabetic complications, animal models

## Abstract

**Context:***Syzygium cumini* (L.) Skeels (Myrtaceae) is a medicinal plant widely used in folk medicine for the treatment of diabetes mellitus (DM). However, studies on the use of this plant and of nanoparticle formulations against DM-related fungal infections are scarce.

**Objective:** To evaluate the effect of the treatments with aqueous seed extract of *S. cumini* (ASc) and ASc-loaded polymeric nanoparticles (NPASc) on biochemical parameters in *Candida albicans*-infected diabetic rats.

**Materials and methods:** Male Wistar rats were divided into eight groups: Control, DM, *C. albicans*, *C. albicans* + ASc, *C. albicans* + NPASc, DM + *C. albicans*, DM + *C. albicans* + ASc and DM + *C. albicans* + NPASc. Rats were daily treated with ASc or NPASc (100 mg/kg) for 21 days. Biochemical parameters in serum and urine, advanced oxidation protein product (AOPP) and TBARS levels in the serum, kidney, liver and pancreas and *N*-acetyl-β-d-glucosaminidase (NAG) activities in kidney and urine were evaluated.

**Results:** Biochemical and oxidative stress parameters increased in rats with DM and/or candidiasis. NPASc was more effective than ASc in decreasing glucose (56%), cholesterol (33%) and creatinine (51%) levels; serum (16%) and pancreatic (46%) AOPP and renal (48%) TBARS levels when compared with DM + *C. albicans* group. In *C. albicans* group, both treatments decreased NAG activity but did not decrease creatinine levels.

**Conclusions:** These data suggest that the use of nanotechnology is able to improve plant extract properties such as antioxidant activity that may be useful in diabetes-related complications.

## Introduction

Diabetes mellitus (DM) is a chronic condition that occurs in response to the lack or reduction of insulin production or by the ineffectiveness of the insulin produced, resulting in increased levels of glucose in the blood. According to the International Diabetes Federation (IDF 2015), there are currently about 415 million diabetic patients, while in 2040 this disease will affect 642 million people. Over time, the resulting high levels of glucose in the blood causes damage to many tissues in the body, leading to the development of disabling and life-threatening health complications (IDF 2015).

Exposure to hyperglycaemia not only causes a decrease in leucocyte function but also leads to a permanent stimulation of polymorphonuclear cells and therefore a less pronounced cellular response to an infective stimulus (Buchta et al. 2013). This is associated with an increased susceptibility of DM patients to fungal infections, such as those caused by *Candida albicans*. During microbial infection, the immune system cells expose the microbes to various insults including reactive nitrogen species (RNS), reactive oxygen species (ROS) and cationic streams (Kaloriti et al. 2014). A lower formation of these free radicals and consequently in this route of microbial elimination is observed in DM (Miramón et al. 2012).

The conventional antifungal therapy has shown many adverse effects, including damage to the liver and kidneys, which may contribute to the aggravation of renal lesions in diabetic patients. In this context, it is necessary to increase the number of studies on the use of natural products with strong pharmacological actions and that can act in the pathophysiology of DM, preventing the development and progression of the complications of this disease. *Syzygium cumini* (L.) Skeels (Myrtaceae) is a plant known for its hypoglycaemic, hypolipidaemic, antifungal, anti-inflammatory and antioxidant pharmacological properties, which are attributed to the presence of bioactive compounds in different parts of the plant, especially the seeds (Ayyanar & Subash-babu 2012; Ayyanar et al. 2013; Srivastava & Chandra 2013). Nevertheless, the therapeutic effect of medicinal plants has been questioned due to very low bioavailability of the main constituents after metabolic transformation by the liver (Conte et al. 2016). Recently, our group demonstrated that polymeric nanoparticles containing an aqueous extract of *S. cumini* were able to maintain the antioxidant properties and to enhance the antifungal activity of the extract *in vitro* (Bitencourt et al. 2016). In particular, polymeric nanoparticles prove to be a valuable alternative, since they can control the release of drugs, are biocompatible, biodegradable and exhibit increased stability when compared to other systems. Considering the growing number of diabetic patients, the recurrent cases of fungal infection in this population and the search for alternative treatments that can alleviate the deleterious effects of DM, this study was aimed at evaluating the effect of treatments with an aqueous seed extract of *S. cumini* (ASc) and ASC-loaded polymeric nanoparticles (NPASc) on biochemical parameters in rats with DM infected or not by *C. albicans.*

## Materials and methods

### Chemicals

Ethyl acetate, methanol, acetonitrile, acetic acid, gallic acid, chlorogenic acid, caffeic acid, and ellagic acid were purchased from Merck (Darmstadt, Germany). Polysorbate 80 (Tween 80^®^), poly-ɛ-caprolactone (PCL), sorbitan monooleate (Span 80^®^), streptozotocin (STZ), catechin, quercetin and rutin reference standards were acquired from Sigma Chemical Co. (St. Louis, MO). All other chemicals were of analytical grade and were obtained from standard commercial suppliers.

### Extract and nanoparticle suspension preparation, characterization and phytochemical analysis

*Syzygium cumini* seeds were collected (29°43′22″S and 53°43′47″W, Santa Maria, Rio Grande do Sul, Brazil) and identified by the Laboratory of Botanic and Pharmacognosy of the Federal University of Santa Maria (voucher number SMDB 14.001). A suspension of 100 g of seeds in 200 mL of distilled water was stirred magnetically overnight (12 h) at room temperature. This was repeated three consecutive times. The residue was removed by filtration and the extract evaporated to dryness at a lower temperature (<40 °C) under reduced pressure in a rotary evaporator. The yield of the extract was 6.2% w/w. The residual extract was dissolved in water for further use (Prince et al. 1998). NPASc were prepared by the emulsification/evaporation solvent method (Quintanar-Guerrero et al. 1998), with modifications according to Bitencourt et al. (2016). Briefly, 10 mg of ASc were dissolved in an aqueous phase containing 1% polysorbate 80. An organic phase (ethyl acetate) containing 1% PCL and 1% sorbitan monooleate was also prepared. After 60 min, the aqueous phase was added to the organic phase, forming the primary emulsion. This emulsion was kept under strong magnetic stirring for 20 min and then a second aqueous phase containing 2% polysorbate 80 was added to the primary emulsion. After 20 min, the emulsion was transferred to a high shear mixer (Marconi, MA-102/PLUS, Piracicaba, Brazil) and stirred at 6000 rpm during 10 min. Then, ethyl acetate was eliminated through rotary evaporation. The total phenolic content present in NPASc (*n* = 3) was determined by diluting an aliquot of the sample in 10 mL acetonitrile followed by 10 min sonication. Samples were filtered and injected into the HPLC system.

The presence of seven antioxidant compounds in ASc and NPASc, namely gallic, chlorogenic, caffeic and ellagic acids, and catechin, quercetin and rutin was investigated by HPLC-DAD. Reverse phase chromatography analyses were carried out under gradient conditions using a C_18_ column (4.6 mm × 150 mm, 5 μm). The mobile phase was composed of water containing 2% acetic acid (A) and acetonitrile (B) until 10 min and changed to obtain 20, 40, 50, 60, 70 and 100% B at 20, 30, 40, 50, 60 and 80 min, respectively, following the method previously described with slight modifications (Boligon et al. 2012). Standards, ASc and NPASc were analyzed at a concentration of 100 μg/mL. Identification of the compounds was performed by comparing their retention times and UV absorption spectra with those of the commercial standards. The flow rate was 0.7 mL/min, injection volume 50 μL and the wavelength were 254 nm for gallic acid and 280 nm for catechin, 327 nm for chlorogenic, ellagic and caffeic acids, and 365 nm for rutin and quercetin. The samples and mobile phase were filtered through 0.45 μm membrane filter (Millipore) and then degassed by ultrasonic bath prior to use. Stock solutions of standard references were prepared in the HPLC mobile phase at a concentration range of 0.030–0.350 mg/mL for quercetin and rutin, 0.040–0.250 mg/mL for gallic, chlorogenic, caffeic and ellagic acids, and 0.025–200 mg/mL for catechin. The chromatography peaks were confirmed by comparing the retention times with those of reference standards and by DAD spectra (200 to 500 nm). Calibration curve for gallic acid: *Y* = 13973*x* + 1095.6 (*r* = 0.9993); catechin: *Y* = 11840*x* + 1178.2 (*r* = 0.9998); epicatechin: *Y* = 12542*x* + 1412.7 (*r* = 0.9991); chlorogenic acid: *Y* = 11864*x* + 1252.8 (*r* = 0.9994); caffeic acid: *Y* = 13178*x* + 1267.2 (*r* = 0.9999); ellagic acid: *Y* = 12681*x* + 1164.9 (*r* = 0.9998); rutin: *Y* = 13077*x* + 1265.4 (*r* = 0.9992); isoquercitrin: *Y*= 11927*x* + 1306.2 (*r* = 0.9996); quercitrin: *Y* = 13470*x* + 1293.7 (*r* = 0.9994); kaempferol: *Y* = 11865x + 1359.5 (*r* = 0.9999) and quercetin: *Y* = 12693*x* + 1176.0 (*r* = 0.9997). All chromatographic operations were carried out at ambient temperature and in triplicate.

### Induction of diabetes, *Candida albicans* infection and experimental design

Male albino Wistar rats (weighing 180 ± 10 g) were reared, before and after treatment, under similar environmental conditions, with food and drinking water *ad libitum*. In this study, the animals were divided into eight groups (*n* = 6) as follows:

Control rats;

DM: diabetic rats;

CA: rats infected with *C. albicans*;

CA + ASc: rats infected with *C. albicans* and treated with ASc;

CA + NPASc: rats infected with *C. albicans* and treated with NPASc;

DM + CA: diabetic rats infected with *C. albicans*;

DM + CA + ASc: diabetic rats infected with *C. albicans* and treated with ASc;

DM + CA + NPASc: diabetic rats infected with *C. albicans* and treated with NPASc.

Type 1 DM was induced in the rats by intra-peritoneal injection of streptozotocin at a dose of 60 mg/kg (Bitencourt et al. 2015). Only diabetic rats with a fasting blood glucose level of at least 250 mg/dL were included in the experiment. Fifteen days after DM induction, rats in groups 3–8 were inoculated intraperitoneally with 0.2 mL of a suspension containing 10^5^ colony forming units (CFU)/mL obtained from a clinical *C. albicans* strain (Fisher et al. 1989).

ASc and NPASc were administered by gavage at a dose of 100 mg/kg (Bitencourt et al. 2015). At day 21 of experiment, the rats were anesthetized with isoflurane, killed by decaptation and blood and urine were collected. Samples of liver, kidneys and pancreas were rapidly dissected, weighed and homogenized in appropriate buffer.

All animal experiments were conducted in accordance with principles for laboratory animal use and care, as described in the guidelines of the Ethics Committee for Animal Research of the Federal University of Santa Maria, which approved the experimental protocol (n° 074/2014).

### Biochemical analysis

Serum obtained by centrifugation was analyzed spectrophotometrically for glucose, fructosamine, total protein, cholesterol, triacylglycerol and creatinine levels using commercial diagnostic kits (Labtest Diagnóstica, Lagoa Santa, Brazil). The hepatic glycogen content was determined by the method previously described (Krisman 1962) and the results were expressed as mg of glycogen per mg of tissue.

### Oxidative stress levels

The protein oxidation was assessed through measurement of advanced oxidation protein product (AOPP) concentrations in serum and tissues (Witko-Sarsat et al. 1998). Lipid peroxidation was estimated in serum and tissues by measurement of thiobarbituric acid reactive substances (TBARS) (Buege & Aust 1978; Ohkawa et al. 1979). The protein concentration was measured by the method of Lowry et al. (1951), using bovine serum albumin as the standard.

### *N*-Acetyl-β-d-glucosaminidase (NAG) assay and urinary parameters

Kidney homogenates and urine samples were added to an enzyme reaction mixture that consists of a substrate (*p*-nitrophenyl-*N*-acetyl-β-d-glucosaminide) dissolved in sodium citrate buffer (pH 4.4). Samples were incubated at 37 °C for 15 min, and the reaction was stopped by adding 2-amino-2-methyl-1-propanol buffer (pH 10.25). NAG activity is proportional to the absorbance of the liberated *p*-nitrophenylate ion measured spectrophotometrically at 405 nm after correction of absorbance using a urine blank sample (Horak et al. 1981). The protein concentration was measured by the method of Lowry et al. (1951). Urinary glucose levels and total protein were determined using commercial diagnostic kits (Labtest Diagnóstica, Lagoa Santa, Brazil).

### Statistical analysis

Data were analyzed by one-way analysis of variance (ANOVA) followed by Duncan’s *post hoc* test using Statistica 6.0 software (StatSoft. Inc., Tulsa, OK). The limit of statistical significance was set at *p* < 0.05. The results were expressed as mean ± SEM.

## Results and discussion

Efforts have been made to develop nanoparticles with physical, chemical and biological biocompatible properties that can be applied to optimize and overcome limitations on the use of medicinal plants, improving solubility and increasing the rate of release penetration, and distribution of vegetal extracts (Mora-Huertas et al. 2010; Bonifácio et al. 2014; Rezaei-kelishadi et al. 2014). ASc showed high concentrations of phenols and flavonoids (gallic acid: 10.79 mg/g; catechin: 2.25 mg/g; chlorogenic acid: 5.62 mg/g; caffeic acid: 2.65 mg/g; ellagic acid: 2.08 mg/g; rutin: 7.76 mg/g; quercetin: 2.78 mg/g), which were not altered in NPASc (gallic acid: 10.16 mg/g; catechin: 2.23 mg/g; chlorogenic: 5.44 mg/g; caffeic acid: 2.18 mg/g; ellagic acid: 2.02 mg/g; rutin: 7.60 mg/g; quercetin: 2.75 mg/g). The development of NPASc was successful, as already previously demonstrated (Bitencourt et al. 2016).

As expected, animals with DM (DM and DM + CA groups) showed significantly increased serum levels of glucose, fructosamine, cholesterol, triglycerides and creatinine, and significantly decreased levels of protein and hepatic glycogen (Table 1), when compared to Control. The treatment of diabetic animals infected with *C. albicans* with NPASc (DM + CA + NPASc) achieved better therapeutic effect than the treatment with ASc (DM + CA + ASc) in attenuating total cholesterol and serum glucose levels, when compared to DM + CA group. The hypoglycaemic and hypolipidaemic action of the ASc has been shown in the literature (Ayyanar et al. 2013; Bitencourt et al. 2015). In addition, the main compounds found in ASc and NPASc, gallic acid, chlorogenic acid and rutin have been studied for their hypoglycaemic activity in rat models of DM (Meng et al. 2013; Kade et al. 2014; Saklani et al. 2016). Nonetheless, the superior effects observed for NPASc are likely to be related to the physicochemical properties of these structures such as greater protection against oxidation and other degrading reactions that occur in the initial segments of intestine, where the phenolic substances present in the extract have major absorption (Ferriz & Vinová 2010; Bonifácio et al. 2014).

**Table 1.** Effect of the treatment with ASc and NPASc in biochemical parameters in rats.

	Control	DM	CA	CA + ASc	CA + NPASc	DM + CA	DM + CA + ASc	DM + CA + NPASc
Serum								
Glucose	83.2 ± 2.6b	358.2 ± 18.9a	83.7 ± 6.9b	90 ± 4.5b	90.5 ± 1.9b	346.5 ± 15a	180.2 ± 8.7c	153.1 ± 8.8d
Fructosamin	0.9 ± 0.1b	2.1 ± 0.1a	0.9 ± 0.1b	0.9 ± 0.1b	1.1 ± 0.1b	2 ± 0.3a	1.1 ± 0.1b	1 ± 0.1b
Cholesterol	86.8 ± 2.2b	107.4 ± 4a	85.2 ± 1.9b	79.3 ± 5.4b	76.4 ± 3.4b	111.8 ± 5.7a	89.1 ± 3.1b	74.7 ± 5.7c
Triglycerides	80.6 ± 3.4b	162.1 ± 9.4a	86.3 ± 3.1b	90.6 ± 3.2b	80.2 ± 6b	151.5 ± 3a	90.5 ± 5.8b	90.2 ± 4.3b
Creatinine	0.3 ± 0.1d	0.6 ± 0.1a	0.5 ± 0.1bc	0.4 ± 0.1c	0.4 ± 0.1bc	0.5 ± 0.1ab	0.3 ± 0.1d	0.2 ± 0.1d
Protein	6.9 ± 0.3a	5.4 ± 0.2b	6.9 ± 0.2a	6.5 ± 0.3a	6.5 ± 0.3a	5.8 ± 0.2b	6.6 ± 0.4a	6.9 ± 0.3a
Hepatic glycogen	1.2 ± 0.1b	0.8 ± 0.1e	1.1 ± 0.1b	1.1 ± 0.1b	1.2 ± 0.1b	0.9 ± 0.1c	1.6 ± 0.1a	1.8 ± 0.2a
Urine								
Glucose	4.5 ± 0.4c	460.5 ± 14.4a	4.5 ± 0.6c	4.7 ± 0.3c	5.1 ± 0.6c	429.7 ± 26.7a	211.9 ± 11.1b	215.0 ± 9.4b
Protein	88.2 ± 8.3ce	134.8 ± 8.6b	82.9 ± 3.8ce	71.1 ± 6.1c	78.4 ± 9.3c	181.2 ± 8a	93.3 ± 6.7de	84.1 ± 10e
Urinary NAG	0.4 ± 0.04e	3.6 ± 0.4c	1.3 ± 0.1d	0.9 ± 0.1de	0.6 ± 0.1e	7.6 ± 1.1a	5.7 ± 0.3b	4 ± 0.4c
Kidney NAG	549.6 ± 23e	1306 ± 13a	1234 ± 37a	834.9 ± 27d	800.4 ± 31d	1120 ± 25a	998.7 ± 30c	946.8 ± 23c

Values are expressed as mean ± SEM (*n* = 6). Mean values with different letters in a row differ (*p* < 0.05) by the Duncan test. DM: diabetic rats; CA: rats infected with *C. albicans*; CA + ASc: rats infected with *C. albicans* and treated with ASc; CA + NPASc: rats infected with *C. albicans* and treated with NPASc; DM + CA: diabetic rats infected with *C. albicans*; DM + CA + ASc: diabetic rats infected with *C. albicans* and treated with ASc; DM + CA + NPASc: diabetic rats infected with *C. albicans* and treated with NPASc. Serum: Glucose (mg/dL), Fructosamine (mmol/L), Insulin (ng/mL), Cholesterol (mg/dL), Triglycerides (mg/dL), Creatinine (mg/dL), Protein (g/dL), Hepatic glycogen (mg/mg of tissue); Urine: Glucose (mg/dL), Protein (mg/dL), Urinary NAG (U/L), Kidney NAG (U/L/mg of tissue).

A significant increase in proteinuria, glycosuria and NAG urinary excretion was observed in diabetic animals infected or not by *C. albicans* (DM and DM + CA). It has been demonstrated that DM causes changes in the activity of kidney enzymes, thereby leading to increased excretion of proteins and glucose and culminating in the development of kidney damage (Komala et al. 2014). NAG is a lysosomal brush border enzyme found in the proximal tubular cells and, because of its relatively high molecular weight (>130 kDa), it is not filtered through the glomeruli. However, NAG is released into the urine after renal tubular injury, which justifies its use as a marker of proximal tubular injury of diverse causes (Liangos et al. 2007; Vaidya et al. 2008; Moresco et al. 2013). In addition, previous studies have suggested that this enzyme is also produced by *C. albicans* strains, and is likely to be involved in the pathogenicity of this fungal species, favouring the growth and colonization of cell surfaces (Niimi et al. 1997; Munro et al. 2001). Interestingly, an increase in serum creatinine levels and in kidney and urinary NAG activity was also observed in non-diabetic rats infected with *C. albicans* (CA; Table 1).

In non-diabetic rats infected with *C. albicans*, the treatments with ASc and NPASc were effective in reducing urinary and kidney NAG activity, but did not reduce serum creatinine levels. In diabetic groups infected with *C. albicans*, treatments reduced serum creatinine levels, proteinuria and glycosuria. Indeed, NPASc was more effective than ASc in reducing the levels of urinary NAG activity. Under inflammation, interstitial spaces are wider, allowing the retention of polymeric nanoparticles and the release of the extract to exert its pharmacological actions within the affected tissue (Tian et al. 2012).

**Figure 1.**
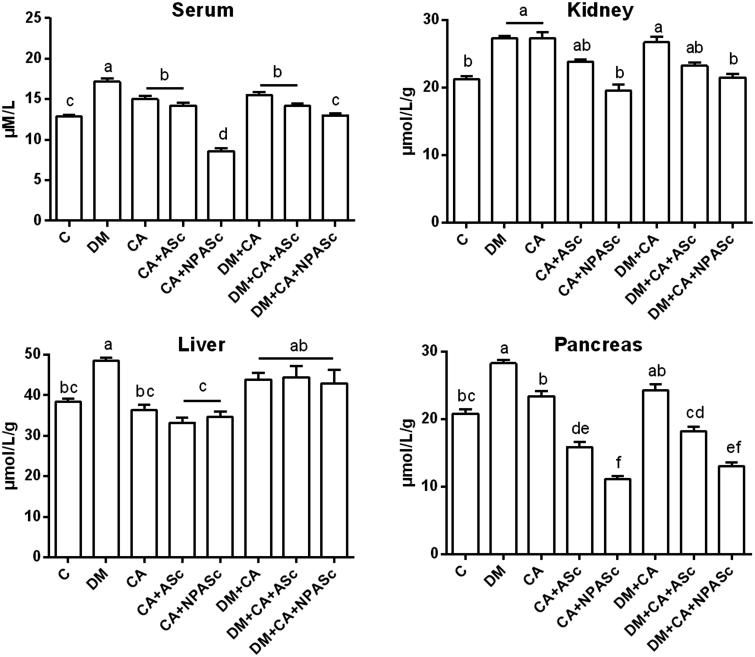
Effect of the treatment with ASc and NPASc on AOPP levels in serum (μM/L) and tissue (μmol/L/g of protein) of rats. Values are expressed as mean ± SEM (*n* = 6). Mean values with different letters differ (*p* < 0.05) by the Duncan test. DM: diabetic rats; CA: rats infected with *C. albicans*; CA + ASc: rats infected with *C. albicans* and treated with ASc; CA + NPASc: rats infected with *C. albicans* and treated with NPASc; DM + CA: diabetic rats infected with *C. albicans*; DM + CA + ASc: diabetic rats infected with *C. albicans* and treated with ASc; DM + CA + NPASc: diabetic rats infected with *C. albicans* and treated with NPASc.

Oxidative stress in DM increases non-enzymatic protein glycation forming advanced glycation end-products (AGEs) and a class of compounds with physicochemical characteristics similar to AGEs, named AOPP (Selmeci 2011). In the present study, we observed an increase in the AOPP levels in serum and kidney in DM, CA and DM + CA groups when compared to Control (Figure 1). The increase in AOPP formation is related to the degree of oxidative modifications in proteins and to inflammation, in line with the results of kidney NAG activity. A significantly increase in AOPP levels was also observed in the liver and pancreas of the DM group in comparison to Control. The increase in AOPP levels has been associated with DM, including in animal models, and may influence the development of chronic inflammatory complications of this pathology (Lee et al. 2010; Tiwari et al. 2014; Badawy et al. 2015; Koroglu et al. 2015). NPASc treatments (CA + NPASc and DM + CA + NPASc) had superior effect (*p* < 0.01) when compared to ASc (*p* < 0.05; CA + ASc and DM + CA + NPASc) in decreasing the AOPP levels in serum and pancreas, when compared to respective controls (CA and CA + DM). Most of *S. cumini* active principles such as flavonoids are highly soluble in water and have poor-lipid solubility. These characteristics severely limit their ability to cross biological membranes, altering the pharmacodynamic profile of therapeutic compounds. Therefore, the results observed with AOPP may be related to the characteristics of polymeric nanoparticles such as enhancement of solubility, bioavailability, therapeutic index, stability and controlled delivery, as well as to the antioxidant and anti-inflammatory properties of the extract constituents (Rasoanaivo et al. 2011; Conte et al. 2016). Recently, a liposomal formulation was able to improve the oral bioavailability and antioxidant activity of chlorogenic acid (Feng et al. 2016). Moreover, chlorogenic acid-loaded chitosan nanoparticles showed a controlled release profile and a preserved antioxidant activity under *in vitro* conditions (Nallamuthu et al. 2015). Chitosan is a non-toxicity polymer with permeation enhancing properties that is very widely used in combination with PCL. In the same way, a d-α-tocopheryl polyethylene glycol 1000 succinate nanoemulsion showed significant improvement in solubility, *in vitro* release, *ex vivo* permeation and antioxidant activity of rutin (Sharma et al. 2015). A silica nanoparticle presented similar effects for gallic acid (Hu et al. 2013).

**Figure 2.**
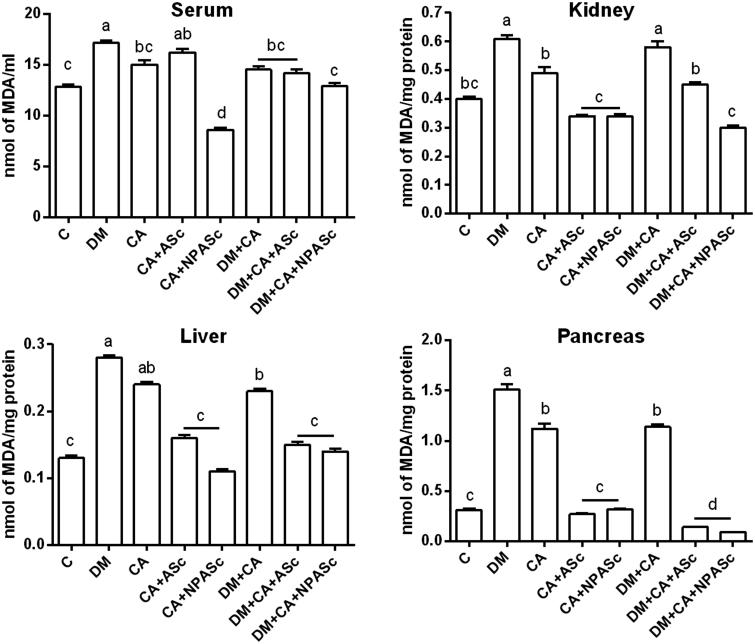
Effect of the treatment with ASc and NPASc on lipoperoxidation levels in serum (nmol of MDA/ml) and tissue (nmol of MDA/mg protein) of rats. Values are expressed as mean ± SEM (*n* = 6). Mean values with different letters differ (*p* < 0.05) by the Duncan test. DM: diabetic rats; CA: rats infected with *C. albicans*; CA + ASc: rats infected with *C. albicans* and treated with ASc; CA + NPASc: rats infected with *C. albicans* and treated with NPASc; DM + CA: diabetic rats infected with *C. albicans*; DM + CA + ASc: diabetic rats infected with *C. albicans* and treated with ASc; DM + CA + NPASc: diabetic rats infected with *C. albicans* and treated with NPASc.

TBARS levels in the kidney, liver and pancreas showed a similar, significant increase in DM, CA and DM + CA groups when compared to Control (Figure 2). This shows that both the infection and the DM promote oxidative stress but do not act synergistically. Nonetheless, despite the production of free radicals is a mechanism by which macrophages kill pathogens, systemic oxidative stress is not considered a threat to the pathogen (Enjalbert et al. 2007). Therefore, attenuating this process would benefit the host against the deleterious effects of oxidative stress. ASc and NPASc treatments were able to decrease TBARS levels in the treated groups (CA + ASc, CA + NPASc, DM + CA + NPASc, DM + CA + NPASc) when compared to respective control groups (CA and DM + CA). The high antioxidant power of *S. cumini* is attributed to the presence of phenols and flavonoids, which helps to counteract the pro-oxidant response of the host (Ayyanar et al. 2013).

## Conclusions

The present study demonstrated that diabetes and/or *C. albicans* infection alter biochemical parameters in serum, urine and tissues of rats. The evaluated parameters indicated that NPASc was more effective than ASc improving the antioxidant properties of the extract and ameliorating oxidative burst evoked by DM and/or candida infection in rats. Therefore, ASc and NPASc could be used as an adjunctive therapy for chronic complications of DM.
